# A Truncated Mutation of TP53 Promotes Chemoresistance in Tongue Squamous Cell Carcinoma

**DOI:** 10.3390/ijms26052353

**Published:** 2025-03-06

**Authors:** Xiaoyun Du, Junheng Zheng, Xiangwan Lu, Yan Zhang

**Affiliations:** MOE Key Laboratory of Gene Function and Regulation, School of Life Sciences, Sun Yat-sen University, Guangzhou 510275, China; duxy29@mail2.sysu.edu.cn (X.D.); zhengjunheng@gmail.com (J.Z.); luxiangw@mail.sysu.edu.cn (X.L.)

**Keywords:** TSCC, TP53 mutation, ID2, NER, chemoresistance, cancer stem cells

## Abstract

Tongue squamous cell carcinoma (TSCC), a subtype of head and neck squamous cell carcinoma, is characterized by frequent chemoresistance. Genetic mutations commonly observed in TSCC play a critical role in malignant progression; thus, elucidating their functional significance is essential for developing effective treatment strategies. To more accurately investigate the relationship between mutations and chemoresistance, we established low-passage TSCC cells, CTSC-1, obtained from a chemoresistant patient, and CTSC-2, from a treatment-naïve patient. Sanger sequencing revealed a specific TP53 mutation (Q331*) in CTSC-1, leading to the loss of the tetramerization and C-terminal regulatory domains. Notably, CTSC-1 cells harboring TP53-Q331* and CTSC-2 cells with TP53 knockout that have been engineered to ectopically express TP53-Q331* exhibit enhanced chemoresistance and increased cancer stem cell-like properties. Mechanistically, TP53-Q331* upregulates the expression of inhibitor of DNA binding 2 (ID2), which is crucial for maintaining the stemness of TSCC cells. Subsequently, ID2 activates the expression of nucleotide excision repair (NER) pathway-related genes ERCC4 and ERCC8, thereby enhancing the chemoresistance in TSCC. In conclusion, our study demonstrates that the TP53-Q331* mutation enhances TSCC chemoresistance through an ID2-mediated NER pathway, providing a potential prognostic marker and therapeutic target for TSCC chemotherapy resistance.

## 1. Introduction

Head and neck squamous cell carcinoma (HNSCC) ranks as the sixth most common cancer globally, with high incidence and mortality rates [1]. Tongue squamous cell carcinoma (TSCC), the most common subtype of HNSCC, is a significant global health issue, accounting for a substantial number of new cases and deaths annually. Despite advancements in multimodal therapies, the 5-year survival rate for TSCC has stagnated at around 50%, primarily due to intrinsic or acquired chemoresistance and recurrence [2,3].

TSCC is characterized by a range of genetic mutations that drive its development and progression. Among the most commonly observed mutations are those in tumor protein p53 (TP53), notch receptor 1 (NOTCH1), caspase 8 (CASP8), and cyclin-dependent kinase inhibitor 2A (CDKN2A) [4,5]. TP53 gene mutations are among the most frequently observed in TSCC, with reported mutation rates varying between 27.7% and 60% [6]. TP53 encodes wild-type form TP53 protein (TP53-WT) consisting of 393 amino acids. The TP53 protein contains several key functional domains, including the N-terminal transactivation domain (TAD), the central DNA-binding domain (DBD), the tetramerization domain (TD), and the C-terminal regulatory domain (C-Reg) [7]. The TAD domain is responsible for transcriptional activation, the DBD domain is crucial for DNA binding and transcriptional activation, the TD domain aids in tetramer formation, and the C-Reg domain is important for stabilizing tetramers that bind DNA with high affinity and recruit transcriptional cofactors. TP53-WT regulates gene expression in response to stress and serves as a tumor suppressor by promoting DNA repair, regulating the cell cycle, and inducing apoptosis [8,9]. TP53-WT also maintains the balance between self-renewal and differentiation in normal cells and CSCs [10]. The status of TP53 in tumors significantly influences chemotherapy efficacy and tumor progression. Mutation can result in the loss or alteration of TP53 function [11,12]. The majority of common TP53 mutations are located in exons 5–8, which encode the DBD domain [13]. Missense mutations represent the most frequent type of TP53 mutation, typically leading to a loss of tumor-suppressing function and acquisition of oncogenic properties [14]. Nonsense mutations produce non-functional TP53 proteins and are associated with poorer clinical outcomes [15]. Frameshift mutations similarly result in non-functional TP53 proteins [16]. Hotspot mutations, such as those at codons R175, G245, R248, R273, and R282, frequently affect the DNA-binding region of TP53 [17]. However, certain aspects of TP53 mutations remain poorly understood, particularly regarding mutations that cause partial loss of function or alterations in the tetramer structure. Elucidating the functional consequences of tetramer destabilization is essential, given its role in DNA binding and transcriptional regulation. Current therapies aim to restore TP53-WT function or exploit vulnerabilities linked to TP53 mutations [12].

Chemoresistance in cancer is a complex, multifactorial phenomenon driven by various mechanisms enabling cancer cells to evade the effects of chemotherapeutic agents. Key mechanisms include acquired mutations, the presence of cancer stem cells (CSCs), epithelial–mesenchymal transition (EMT), enhanced drug efflux via ABC transporters, and the activation of DNA damage response pathways [18,19,20]. CSCs, also referred to as tumor-initiating cells, drive tumor heterogeneity, chemoresistance, and recurrence [21,22]. By entering a quiescent or dormant state, CSCs become less susceptible to therapies targeting proliferating cells [23]. The enhanced DNA repair capacity allows CSCs to survive DNA-damaging treatments [24,25]. Understanding how CSCs evade therapies is crucial for developing more effective treatments.

In vitro studies are crucial for understanding the relationship between tumors and chemoresistance. These studies typically use various cell models to investigate the mechanisms underlying chemoresistance and to screen potential therapeutic agents. While long-term cultured cell lines are essential for scientific research, their genomic instability and accumulated mutations can alter cellular traits and resistance profiles, leading to discrepancies with primary tumors [26]. This instability compromises their reliability in resistance research, while low-passage cells are typically without such mutations [27].

In this study, we established and utilized low-passage CTSC-1 and CTSC-2 cells derived from patients with different prognoses to elucidate more precisely the mechanisms underlying tumor gene mutations and expression profiles associated with chemoresistance and recurrence in TSCC. Our findings reveal a previously unrecognized mechanism by which truncating mutations in the TP53 TD domain drive lethal cancer phenotypes. This study highlights the critical role of the TP53-Q331*-ID2-ERCC4/8 axis in promoting chemoresistance in TSCC harboring the TP53-Q331* mutation. Targeting the NER-related gene ERCC4, alongside TP53 gene therapy, may present a potential therapeutic strategy for TSCC.

## 2. Results

### 2.1. Establishment of CTSC-1 and CTSC-2

**Figure 1:**
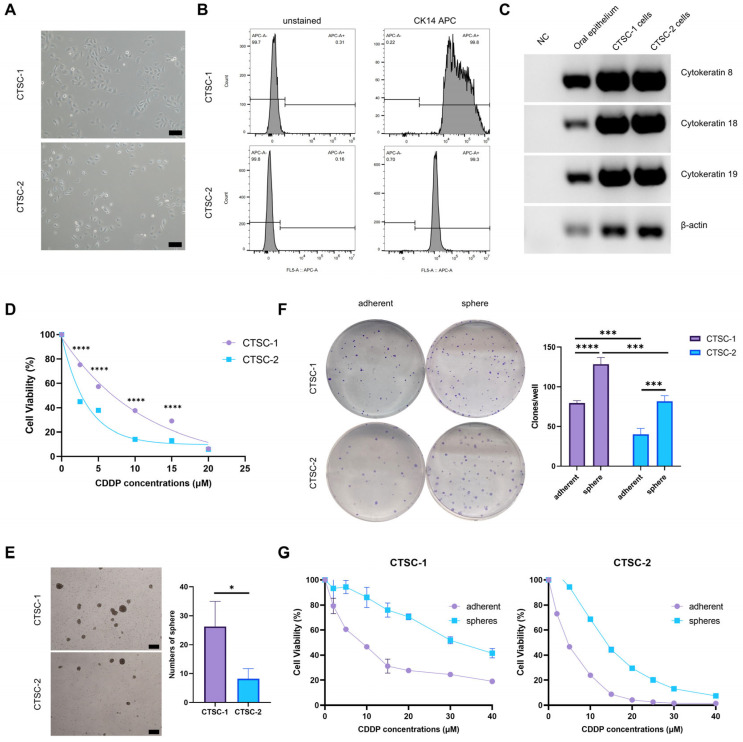
Establishment and characterization of CTSC-1 and CTSC-2 cells. (**A**) Morphology of CTSC-1 and CTSC-2 cells. Scale bars = 200 µm. (**B**) Flow cytometry sorting of CK14-positive squamous epithelial tumor cells. (**C**) RT-PCR analysis of epithelial markers CK8, CK18, and CK19 mRNA expression in CTSC-1 and CTSC-2 cells. (**D**) Dose–response curves of CTSC-1 and CTSC-2 cells treated with CDDP for 48 h. (**E**) Representative images and quantification of neospheres formed by CTSC-1 and CTSC-2 cells after 6 days of culture. Scale bars = 200 µm. (**F**) Colony formation assay to investigate the self-renewal ability of adherent CTSC-1 and CTSC-2 cells and sphere CTSC-1 and CTSC-2 cells. (**G**) Dose–response curves of adherent CTSC-1 and CTSC-2 cells and sphere cells treated with CDDP for 48 h. * *p* < 0.05, *** *p* < 0.001, **** *p* < 0.0001.

To further investigate the relationship between tumor gene expression profiles and chemoresistance, we first cultured two low-passage TSCC cells derived from surgically resected specimens. CTSC-1 was derived from a patient who experienced rapid recurrence after neoadjuvant chemotherapy, while CTSC-2 was derived from a treatment-naïve patient with locally advanced disease. CTSC-1 and CTSC-2 cells grew as adherent monolayers and displayed squamous epithelial morphology (Figure 1A). STR identification confirmed that the genetic information of both CTSC-1 and CTSC-2 cells were consistent with that of their source specimens (Supplementary Table S1). To avoid contamination by other cell types, CK14-positive squamous epithelial tumor cells were isolated via flow cytometry (Figure 1B). Both CTSC-1 and CTSC-2 cells expressed epithelial markers CK8, CK18, and CK19 (Figure 1C). Furthermore, animal experiments demonstrated that both CTSC-1 and CTSC-2 cells could form tumors in nude mice. The xenografts formed by CTSC-1 and CTSC-2 cells resembled their source specimens in morphology (Figure S1A,B).

### 2.2. CTSC-1 and CTSC-2 Have Different Cellular Characteristics

CTSC-1 and CTSC-2 cells exhibited different levels of chemosensitivity, likely due to their distinct origins. Specifically, CTSC-1 cells demonstrated greater resistance to cisplatin (CDDP) than CTSC-2 cells (Figure 1D). Chemoresistance is a multifactorial phenomenon, with CSCs being an important contributor [22,24]. Our previous studies have demonstrated that culturing cancer cells in serum-free medium induces dedifferentiation, leading to the formation of neospheres that exhibit characteristics similar to those of CSCs [28]. CTSC-1 and CTSC-2 cells formed neospheres in serum-free medium with 8F (Figure 1E), with CTSC-1 showing greater neosphere formation, indicating higher self-renewal capacity. Compared to adherent cells, both CTSC-1 and CTSC-2 neospheres exhibited elevated expression of the human embryonic stem cell-specific surface marker, sex-determining region Y-box 2 (Sox2) (Figure S2A). Furthermore, neospheres derived from CTSC-1 and CTSC-2 cells exhibited the ability to differentiate into adipocytes in vitro (Figure S2B), thereby validating their CSC characteristics. Neospheres also exhibited a stronger ability to form colonies compared to adherent cells (Figure 1F). Notably, when comparing adherent cells to neospheres, it was evident that neospheres originating from both CTSC-1 and CTSC-2 cells showed greater resistance to chemotherapy (Figure 1G).

The results demonstrated that both CTSC-1 and CTSC-2 cells are capable of forming neospheres with CSC characteristics. Additionally, CTSC-1 cells showed higher neosphere-forming efficiency, higher expression levels of stem cell markers, enhanced differentiation potential, and stronger colony-forming ability relative to CTSC-2 cells. These findings suggest that CTSC-1 cells display more pronounced CSCs traits compared to CTSC-2 cells, as well as greater resistance to chemotherapy.

### 2.3. A Special TP53 Mutation in CTSC-1

**Figure 2:**
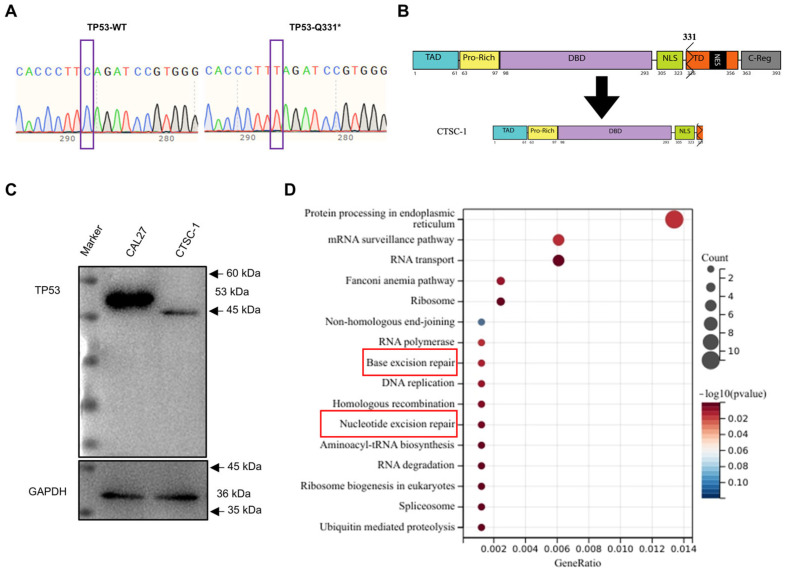
Distinct TP53 mutation and gene expression patterns in CTSC-1 and CTSC-2 cells. (**A**) Sanger sequencing confirming TP53 mutation in CTSC-1 cells. (**B**) Schematic representation of TP53-WT and TP53-Q331*. (**C**) Western blot analysis of TP53 protein expression in CTSC-1 cells using N-terminal antibody. The full-length TP53 expressed in CAL27 cells was used as a control. GAPDH served as a loading control. (**D**) Conducting pathway enrichment analysis of CTSC-1 harboring TP53-Q331* vs. CTSC-2 by KEGG.

To elucidate whether the disparities in chemoresistance and stemness between CTSC-1 and CTSC-2 cells can be attributed to the common mutations observed in TSCC, we performed targeted analysis of TSCC driver genes by RNA-seq and Sanger sequencing. Notably, our findings revealed a distinct TP53 mutation in CTSC-1 cells. Several other common TSCC driver genes exhibited identical mutation types in both CTSC-1 and CTSC-2 cells (Figure 2A and Supplementary Table S2). Specifically, CTSC-1 exhibited a nonsense mutation (Q331*) within exon 10 of TP53, leading to a premature stop codon and truncation of the TD domain, thereby causing the loss of TD and C-Reg domains (Figure 2B).

Western blot analysis using a TP53 antibody confirmed that CTSC-1 cells express a truncated TP53 protein of approximately 45 kDa, retaining the amino terminus and DBD (Figure 2C). This antibody detects endogenous levels of total TP53 protein, with its binding mapped to the amino terminus of the human TP53 protein. TP53 is a key gene that regulates essential cellular processes, including DNA repair, cell cycle control, and other functions, by modulating downstream gene transcription. To investigate the effects of the TP53-Q331* mutation on the transcriptional regulation of genes related to chemoresistance, we performed RNA sequencing to compare the gene expression profiles between CTSC-1 and CTSC-2 cells (Figure S3). Kyoto Encyclopedia of Genes and Genomes (KEGG) pathway enrichment analysis revealed several pathways linked to chemoresistance that were preferentially activated in CTSC-1, including nucleotide excision repair pathway (NER) and base excision repair pathway (Figure 2D). The NER pathway plays a crucial role in removing bulky DNA adducts induced by chemotherapeutic agents, including CDDP. Upregulation of NER activity has been reported to enhance chemoresistance by efficiently repairing DNA damage caused by chemotherapeutic drugs [29,30]. 

These results suggest that the distinct TP53 mutation in CTSC-1 may result in differential DNA damage repair capacities, which subsequently contribute to the observed differences in chemoresistance between CTSC-1 and CTSC-2 cells.

### 2.4. TP53-Q331* Induces Enhanced Chemoresistance and Stemness

**Figure 3:**
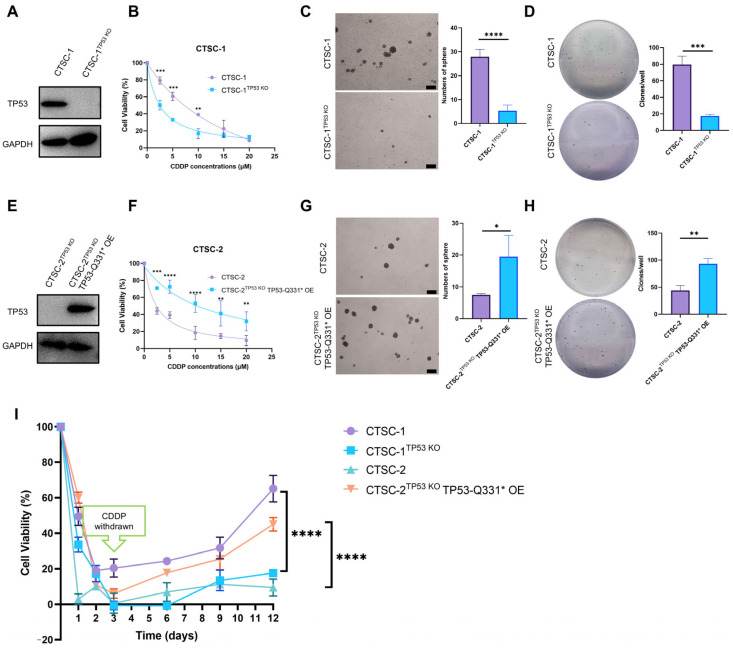
Expression of TP53-Q331* induces enhanced chemoresistance and stemness in CTSC-1 and CTSC-2 cells. (**A**) Western blot verified the knockout of TP53-Q331* in CTSC-1. (**B**) Dose–response curves of CTSC-1 and CTSC-1^TP53 KO^ cells treated with CDDP for 48 h. (**C**) Representative images and quantification of neospheres formed by CTSC-1 and CTSC-1^TP53 KO^ cells after 6 days of culture. Scale bars = 200 µm. (**D**) Colony formation assay to investigate the self-renewal ability of CTSC-1 and CTSC-1^TP53 KO^ cells. (**E**) Western blot verified the overexpression of TP53-Q331* in CTSC-2^TP53 KO^ cells. (**F**) Dose–response curves of CTSC-2 and CTSC-2^TP53 KO^ TP53-Q331* OE cells treated with CDDP for 48 h. (**G**) Representative images and quantification of neospheres formed by CTSC-2 and CTSC-2^TP53 KO^ TP53-Q331* OE cells after 6 days of culture. Scale bars = 200 µm. (**H**) Colony formation assay to investigate the self-renewal ability of CTSC-2 and CTSC-2^TP53 KO^ TP53-Q331* OE cells. (**I**) Cell viability of TSCC cells before and after CDDP treatment. * *p* < 0.05, ** *p* < 0.01, *** *p* < 0.001, **** *p* < 0.0001.

To determine whether the differences in chemoresistance, stemness, and gene expression can be attributable to TP53-Q331* activity, we employed CRISPR/Cas9-mediated gene editing to generate TP53 knockout CTSC-1 and CTSC-2 cells (CTSC-1^TP53 KO^ and CTSC-2^TP53 KO^) (Figure 3A,E). The TP53 knockout resulted in significantly reduced chemoresistance, sphere-forming efficiency, and colony-forming ability in CTSC-1 cells (Figure 3B–D). In contrast, ectopic expression of TP53-Q331* in CTSC-2^TP53 KO^ cells (Figure 3E) was sufficient to enhance chemoresistance, sphere-forming efficiency, and colony-forming ability in CTSC-2 cells (Figure 3F–H), highlighting the crucial role of TP53-Q331* in driving chemoresistance and stemness in CTSC-1 and CTSC-2.

Furthermore, upon exposure to high concentrations of the chemotherapeutic CDDP, we observed that the majority of CTSC-1 and CTSC-2^TP53 KO^ TP53-Q331* OE cells expressing TP53-Q331* initially underwent cell death. However, following the removal of CDDP on day 3, the surviving cells resumed proliferation. In contrast, CTSC-2 and CTSC-1^TP53 KO^ cells exhibited near-total mortality, with significantly slower recovery of proliferation after CDDP removal (Figure 3I). These findings align with the clinical manifestations observed in CTSC-1 patients, where the presence of TP53-Q331* contributes to tumor chemoresistance and recurrence.

Collectively, the above data suggest that TP53-Q331* may be an important factor driving chemoresistance and stemness in TSCC.

### 2.5. ID2 Contributes to TP53-Q331*-Mediated Cell Chemoresistance and Stemness

**Figure 4:**
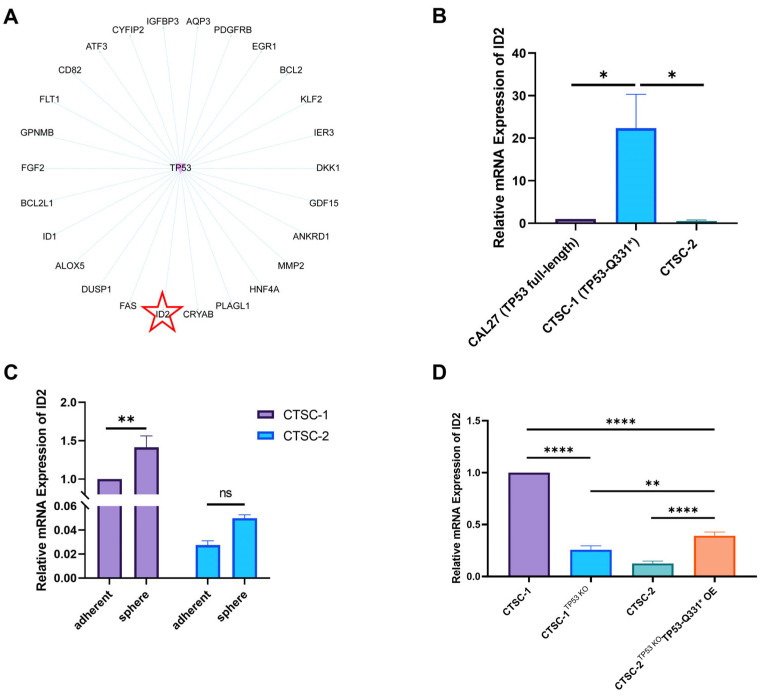
ID2 was a target of TP53-Q331*. (**A**) The GO-TRRUST analysis identified a distinct cohort of genes specifically regulated by TP53 activity. (**B**) qRT-PCR analysis of ID2 mRNA expression level in CTSC-1 and CTSC-2 cells. (**C**) qRT-PCR analysis of ID2 mRNA expression level in adherent CTSC-1 and CTSC-2 cells and sphere cells. (**D**) qRT-PCR analysis shows the correlation between ID2 mRNA expression level and TP53-Q331*. * *p* < 0.05, ** *p* < 0.01, **** *p* < 0.0001.

**Figure 5:**
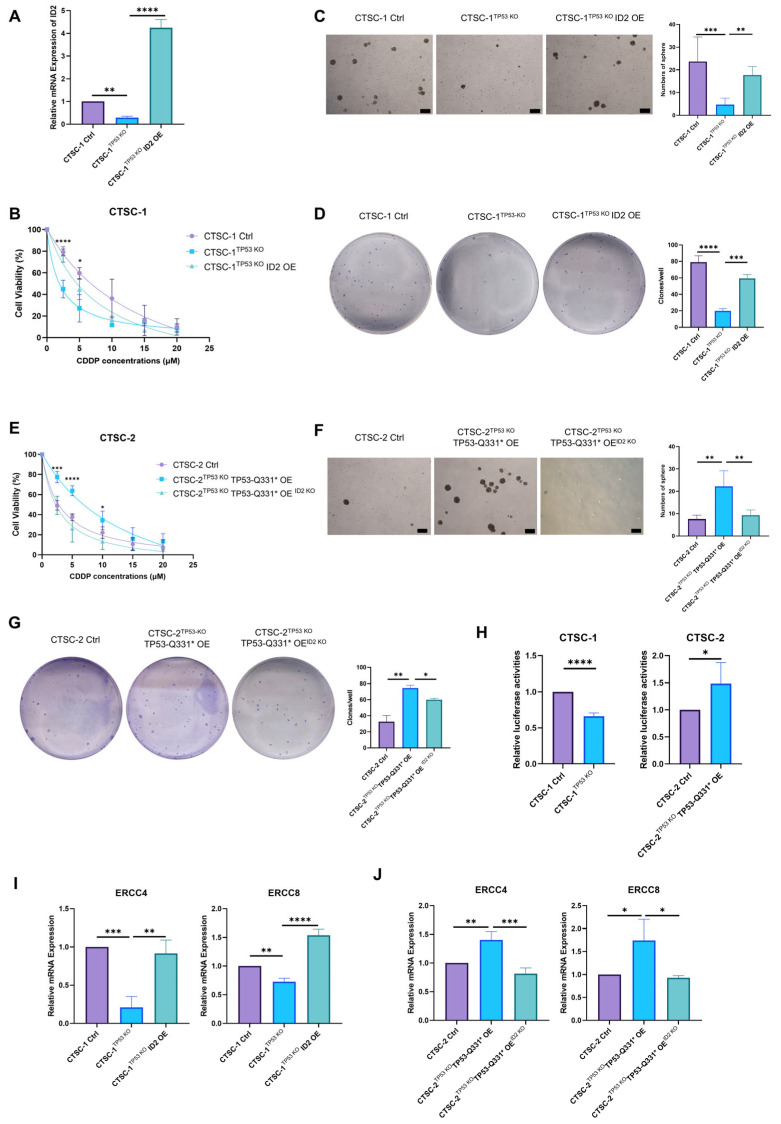
TP53-Q331* mediates cell chemoresistance and stemness through the TP53-Q331*-ID2-ERCC4/8 axis. (**A**) Ectopic expression of ID2 in CTSC-1^TP53 KO^ cells. (**B**) Dose–response curves of CTSC-1, CTSC-1^TP53 KO^, and CTSC-1^TP53 KO^ ID2 OE cells treated with CDDP for 48 h. (**C**) Representative images and quantification of neospheres formed by CTSC-1, CTSC-1^TP53 KO^, and CTSC-1^TP53 KO^ ID2 OE cells. (**D**) Colony formation assay to investigate the self-renewal ability of CTSC-1, CTSC-1^TP53 KO^, and CTSC-1^TP53 KO^ ID2 OE cells. (**E**) Dose–response curves of CTSC-2, CTSC-2^TP53 KO^ TP53-Q331* OE, and CTSC-2^TP53 KO^ TP53-Q331* OE^ID2 KO^ cells treated with CDDP for 48 h. (**F**) Representative images and quantification of neospheres formed by CTSC-2, CTSC-2^TP53 KO^ TP53-Q331* OE, and CTSC-2^TP53 KO^ TP53-Q331* OE^ID2 KO^ cells. (**G**) Colony formation assay to investigate the self-renewal ability of CTSC-2, CTSC-2^TP53 KO^ TP53-Q331* OE, and CTSC-2^TP53 KO^ TP53-Q331* OE^ID2 KO^ cells. (**H**) Luciferase reporter assays in CTSC-1 and CTSC-2 cells transfected with ID2 promoter constructs. (**I**) qRT-PCR analysis of ERCC4 and ERCC8 mRNA expression level in CTSC-1 cells. (**J**) qRT-PCR analysis of ERCC4 and ERCC8 mRNA expression level in CTSC-2 cells. Scale bars = 200 µm. * *p* < 0.05, ** *p* < 0.01, *** *p* < 0.001, **** *p* < 0.0001.

To elucidate the mechanisms by which TP53-Q331* mediates chemoresistance and stemness, we sought to identify the transcriptional signature associated with the activity of TP53-Q331*. In comparison to CTSC-2 cells, 797 genes were upregulated and 1366 genes were downregulated in CTSC-1 cells. GO-TRRUST analysis revealed a distinct set of genes specifically regulated by TP53 activity, with 27 genes being transcriptionally regulated as a result of TP53-Q331* expression (Figure 4A). ID2 emerged as potential effector due to its well-established roles in inhibiting differentiation and maintaining self-renewal and multipotency in stem cells [31]. Compared to CAL27 cells expressing full-length TP53 and CTSC-2 cells, a significant upregulation of ID2 expression was observed in CTSC-1 cells harboring TP53-Q331* (Figure 4B). Moreover, ID2 expression was significantly elevated in CTSC-1 spheres compared to adherent cells, further supporting its role as a critical regulator of stemness (Figure 4C). Silencing of TP53-Q331* in CTSC-1 cells led to a marked reduction in ID2 expression, while TP53-Q331* overexpression in CTSC-2^TP53 KO^ cells induced a significant increase in ID2 levels (Figure 4D). Previous studies have shown that both TP53-WT and point mutant TP53 (R273H/P309S/R248W) can directly suppress ID2 expression, thereby affecting the proliferation capacity of tumor cells [32,33]; our findings indicate that TP53-Q331* exerts the opposite effect by enhancing ID2 expression. These results imply that truncation of the TD and C-Reg domains in TP53-Q331* likely compromises its repressive function on ID2, thereby promoting the derepression of ID2 expression. Moreover, ectopic expression of ID2 in CTSC-1^TP53 KO^ cells resulted in enhanced chemoresistance, increased sphere-forming capacity, and improved colony-forming ability (Figure 5A–D). Conversely, knockout of ID2 markedly impaired chemoresistance, sphere-forming efficiency, and colony-forming ability of CTSC-2^TP53 KO^ TP53-Q331* OE cells (Figure 5E–G), mirroring the phenotypic effects observed upon TP53-Q331* knockout in CTSC-1.

To elucidate the molecular mechanisms by which TP53-Q331* and TP53-WT modulate ID2 expression and to determine whether ID2 is directly regulated by TP53-Q331*, we analyzed the promoter region of ID2. Initially, a reporter gene assay was developed to assess the regulation of ID2 expression. Subsequently, reporter gene vectors were transfected into CTSC-1^TP53 KO^ and CTSC-2^TP53 KO^ TP53-Q331* OE cells to evaluate their promoter activity. The relative promoter activity of ID2 was positively correlated with the expression levels of TP53-Q331*, with a concomitant increase observed as TP53-Q331* levels were elevated (Figure 5H). These findings indicate that TP53-Q331* may directly bind to the ID2 promoter, thereby regulating ID2 expression. This strongly implicates ID2 as a downstream target of TP53-Q331*, playing a critical role in TP53-Q331*-mediated chemoresistance and stemness.

### 2.6. TP53-Q331*-ID2-ERCC4/8 Axis Mediates Chemoresistance

After identifying ID2 as a direct mediator of TP53-Q331* activity, we aimed to further elucidate the downstream effectors linking stemness pathway activation to chemoresistance. By cross-referencing TP53-Q331*-regulated genes with established mediators of chemoresistance, we identified two potential candidates, ERCC4 and ERCC8. Bioinformatics analysis also showed a potential ID2–ERCC4 interaction based on the interaction network (Figure S4). Notably, ERCC4 is a structure-specific endonuclease crucial for NER, the primary pathway responsible for removing CDDP-DNA adducts [29]. ERCC8 serves as a cofactor in the transcription-coupled NER pathway [34]. Previous studies have shown that downregulation of key NER proteins, such as ERCC1, can increase the sensitivity of cancer cells to chemotherapeutic agents, including CDDP and mitomycin C, suggesting that targeting NER components could improve chemotherapy efficacy. Furthermore, NER is implicated in chromatin remodeling and interacts with other repair pathways, such as mismatch repair, thereby complicating its role in chemoresistance [35]. Notably, the expression levels of ERCC4 and ERCC8 were significantly reduced in CTSC-1^TP53 KO^ cells, whereas the ectopic expression of ID2 reinstated the mRNA levels of ERCC4 and ERCC8 (Figure 5I). In CTSC-2 cells with ectopic TP53-Q331* overexpression, a marked upregulation of ERCC4 and ERCC8 expression was observed. Knockout of ID2 in CTSC-2^TP53 KO^ TP53-Q331* OE cells resulted in a substantial reduction in the expression of both ERCC4 and ERCC8 (Figure 5J).

Taken together, these findings reveal ERCC4 and ERCC8 as novel downstream effectors within the TP53-Q331*–ID2 signaling axis, playing a critical role in mediating chemoresistance in TSCC.

### 2.7. TP53-Q331* and ERCC4 Might Be Associated with Poor Prognosis

**Figure 6:**
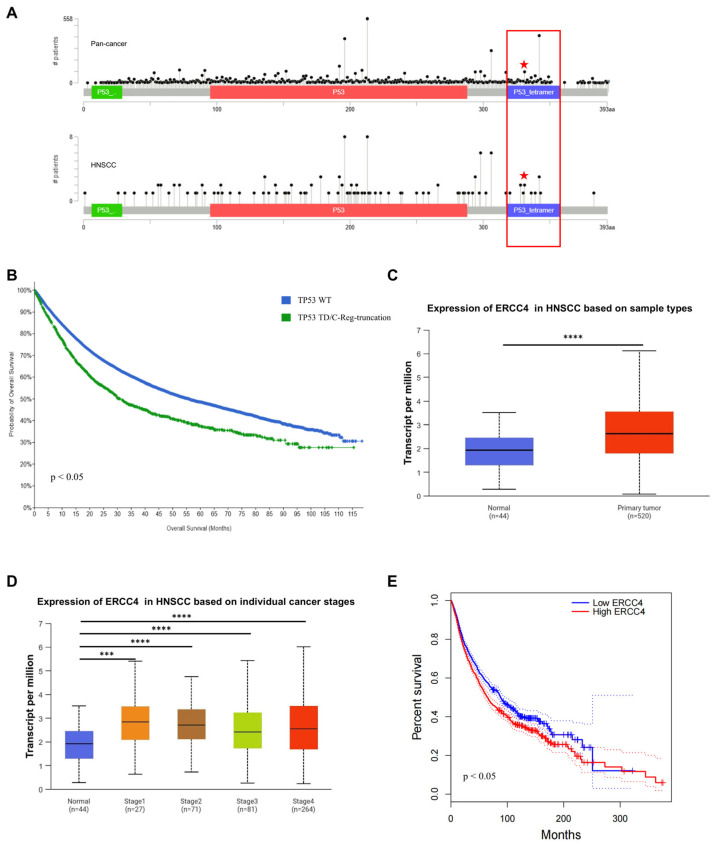
TP53 TD mutations and ERCC4 mRNA expression levels are associated with advanced disease and poor prognosis in HNSCC. (**A**) TP53-Q331* presents in both pan-cancer and HNSCC. Red stars and red box represent TP53-Q331* mutation and mutations occurring on the TD domain, respectively. (**B**) TP53 mutations truncating the TD and C-Reg domains are associated with patient survival outcomes. (**C**) ERCC4 expression in normal and tumor samples. (**D**) ERCC4 expression and cancer stages. (**E**) ERCC4 expression is associated with patient survival outcomes. *** *p* < 0.001, **** *p* < 0.0001.

To further explore the correlation between TP53-Q331* mutation and tumor stage and prognosis, we analyzed its presence in the broader TP53 mutation spectrum and its association with patient survival. TCGA data indicated that the TP53-Q331* mutation is present in a variety of cancers, including HNSCC [36] (Figure 6A). Premature termination mutations resulting in the loss of the TD domain constitute a significant subset of all TP53 mutations. Further TCGA analysis reveals that mutations truncating the TD and C-Reg domains are associated with patient survival outcomes (Figure 6B). To evaluate the clinical significance of our findings, we conducted an analysis of ERCC4 expression using data from TCGA [37]. Our analysis revealed that ERCC4 expression is significantly elevated in tumor samples compared to normal tissues and correlated with cancer progression and prognosis (Figure 6C–E). Additionally, increased ERCC4 expression is associated with CDDP resistance in gastric cancer cells, aligning with our findings [38]. These findings suggest that both TP53-Q331* and ERCC4 might be associated with poor prognosis of HNSCC.

## 3. Discussion

TP53 mutations are frequently observed in various cancers, where this gene serves as a critical tumor suppressor. The spectrum of TP53 mutations encompasses missense mutations, truncating mutations, hotspot mutations, and base transitions [39,40]. In TSCC, TP53 mutations predominantly manifest as missense and truncating mutations. These mutations play a critical role in the pathogenesis and prognosis of TSCC, underscoring the significance of TP53 as both a biomarker and a potential therapeutic target.

Missense mutations are the most prevalent type of TP53 mutation in TSCC. These mutations typically occur in the DBD of the TP53, which is critical for its tumor suppressor function. For example, p.C176F and p.R282W have been identified in TSCC cases [41]. A separate study reported that 43 out of 52 TP53 mutations in TSCC were missense mutations in the DBD [42]. These mutations can result in the loss of tumor suppressor function or confer oncogenic properties, thereby facilitating cancer progression [43]. Truncation mutations lead to the production of a truncated, non-functional TP53 protein. These mutations are also prevalent in TSCC and associated with more aggressive tumor behavior and a poorer prognosis. Hotspot mutations refer to codons within the TP53 gene that exhibit a high mutation frequency. For instance, the Y220C mutation is a significant hotspot in HNSCC, including TSCC [44]. TP53 mutations frequently result in the loss of its tumor suppressor functions, including DNA repair, cell cycle arrest, and apoptosis. The loss of these functions enables cells to proliferate uncontrollably. Therefore, TP53 mutations are associated with poor responses to chemotherapy and radiotherapy, as the mutant TP53 protein fails to induce apoptosis in response to DNA damage caused by these treatments [45]. Compared to patients with TP53-WT, those with TP53 mutations generally exhibit poorer prognosis and reduced overall survival. This is attributed to the aggressive nature of tumors harboring these mutations and their resistance to conventional therapies [46].

Mutations in the TD, including the TP53-Q331* mutation identified in CTSC-1, have remained largely unexplored. While the TP53-Q331* mutation has been reported in adenosquamous carcinoma of the gallbladder and breast cancer, the precise relationship between TP53-Q331* and tumor progression, as well as its role in pathogenesis, remain unclear [47,48]. The biological and clinical significance of TP53 TD mutations has long been overlooked, partly due to their lower frequency compared to hotspot mutations in the DBD. Our study provides a comprehensive analysis of the functional effects of recurrent TD truncation mutations in TSCC, for the first time. We demonstrate that TP53-Q331* functionally promotes chemoresistance and stemness. In comparison to the common TP53 DBD missense mutations, TP53-Q331* predominantly abolishes the tumor suppressor activity of TP53. Our study provides novel insights into the mechanism by which TP53 TD mutations regulate chemoresistance and stemness, and clarifies the relationship between TP53-Q331* and tumor progression.

Long-term in vitro cultured cell lines have been extensively utilized in research, owing to their unlimited proliferative capacity and ease of manipulation. However, prolonged culture can introduce substantial limitations that may undermine the reliability and validity of experimental results. Long-term in vitro culture often induces genetic mutations and chromosomal instability, leading to genetic drift. Consequently, these cells may no longer faithfully represent the original tissues or cell types from which they were derived [49,50]. In addition to genetic alterations, phenotypic drift may occur, resulting in modifications in cellular characteristics and behaviors that can influence experimental outcomes [51]. Long-term in vitro culture can also induce epigenetic changes, such as changes in DNA methylation patterns, which in turn affect gene expression and cellular behavior [52,53]. In particular, prolonged culture of certain cell types, including stem cells, can result in the loss of differentiation potential, thereby diminishing their utility for specific research applications [54]. These genetic and phenotypic changes can render data obtained from long-term cultured cell lines irreproducible, potentially leading to erroneous conclusions [50,55].

Utilizing low-passage cells in research confers several critical advantages, primarily in maintaining the integrity and reliability of experimental results. Low-passage cells more closely resemble their original state, thereby minimizing the risk of genetic drift and selective pressures that are often associated with high-passage cells [56]. Consequently, this ensures that the cells retain characteristics analogous to those of the original tissues or tumors, leading to more precise and dependable data [57]. Low-passage cells are appropriate for preclinical drug testing, as they maintain the original tumor’s drug sensitivity and resistance profiles, thereby providing more reliable and predictive data for therapeutic efficacy [57,58]. Low-passage cells ensure higher reproducibility of experimental results due to their uniformity and stability, which is essential for consistent validation across different research groups and experiments [59]. In this study, we confirmed that the isolated low-passage CTSC-1 and CTSC-2 cells retained histomorphological and chemoresistance characteristics closely resembling those of their original clinical specimens. Consequently, low-passage CTSC-1 and CTSC-2 cells represent valuable models for investigating TSCC and chemoresistance. Significantly, in our investigation of the regulatory effect of TP53-Q331* on the expression levels of the downstream gene ID2, we observed that the expression levels of ID2 in CTSC-1 and CTSC-2^TP53 KO^ TP53-Q331* OE cells, which have different genetic backgrounds, were not identical. However, the regulatory trends were consistent across both cells. This observation suggests that cellular heterogeneity may partially influence research outcomes. Variations in genetic backgrounds among cell lines can lead to functional diversity, thereby affecting the generalizability of research findings [60]. Nevertheless, the use of two distinct cell models in our study validates the consistency of the regulatory trends, thereby enhancing the reliability of our research. Despite this, expanding the sample size is of significant importance to improve the reliability and generalizability of the research findings.

CTSC-1 and CTSC-2^TP53 KO^ TP53-Q331* OE cells showed re-proliferation after chemotherapeutic drug treatment. It is suggested that the presence of TP53-Q331* leads to chemoresistance and may be associated with tumor recurrence. Based on these observations, we hypothesize that gene therapy targeting TP53 may provide a potential therapeutic strategy for treating TSCC. Gene therapy is an advanced medical technology that involves the delivery of nucleic acids into the patient’s cells to treat or prevent diseases by correcting genetic defects or modulating gene expression. This therapeutic approach can be employed to replace defective genes, introduce novel genes, and alter gene expression patterns [61]. Gene therapy has shown promise in treating genetic disorders, cancers, and other acquired diseases [62,63]. In addition, the first gene therapy approved for clinical use is TP53-based gene therapy [64]. Currently, compounds that restore TP53-WT function or eliminate mutant TP53 are under development. These methods are highly dependent on the specific structure and type of TP53 mutation [65,66]. Furthermore, combining TP53-based gene therapy with DNA-damaging chemotherapy or radiotherapy has demonstrated synergistic effects in preclinical and clinical studies [64]. The success of combination therapies involving TP53, combined with our findings in this study that TP53-Q331* can regulate the expression of ERCC4 and both TP53-Q331* and ERCC4, are associated with poor prognosis of HNSCC, suggesting a potential synergistic effect between TP53 gene therapy with targeted drugs against the NER pathway. However, current studies indicate that DNA repair activation in clinical settings involves multifactorial regulation, including gene mutations, oxidative stress, and microenvironment-driven genomic instability, all of which correlate with therapeutic prognosis [67,68]. This suggests that the increased expression of ERCC4/8 may also be related to factors other than TP53-Q331* and ID2. This biological complexity underscores the need for rigorous functional validation. Future studies employing direct ERCC4/8 manipulation (e.g., activation or knockdown) should be prioritized to definitively establish their causal roles in chemoresistance. Successful implementation would not only dissect their specific contributions to chemoresistance but also accelerate therapeutic development targeting DNA repair-driven chemoresistance.

In conclusion, our study elucidates the novel role of the TP53-Q331*-truncating mutation in driving TSCC progression and conferring chemoresistance. By dissecting the TP53-Q331*–ID2–ERCC4/8 axis, we have established a mechanistic link among TP53 dysfunction, aberrant stemness, and hyperactivation of the NER pathway, providing new insights into chemoresistance. The combination of TP53 gene therapy with therapies targeting key downstream effectors such as ID2 and NER pathway-related genes associated with TP53-Q331*, as identified in this study, may offer a novel therapeutic strategy for future preclinical and clinical investigations. Further investigation into the intricate mechanisms by which TP53 mutations facilitate tumorigenesis and confer resistance to therapeutic interventions is imperative for advancing treatment strategies and improving clinical outcomes in patients afflicted with this aggressive malignancy.

## 4. Materials and Methods

### 4.1. Primary Cells, Cell Lines, and Culture Conditions

The tissues used in this experiment were all obtained from surgical specimens removed at Guanghua Stomatological Hospital, Sun Yat-sen University. All samples were anonymously coded in accordance with local ethical guidelines, and written informed consent was obtained from the patients providing the samples. The study was ethically approved and performed in accordance with the Declaration of Helsinki. CTSC-1 and CTSC-2 cells were established from surgically resected primary TSCCs. Primary culturing of TSCC cells was performed using the tissue block adherence method. Collagen-coated culture dishes were left at room temperature for more than 1 h. The dishes were then washed with 1 × PBS. The tissues were then disinfected, disaggregated into small clumps, and evenly distributed into the culture dishes. An appropriate amount of DMEM/F12 culture medium (DF12; Sigma-Aldrich, St. Louis, MO, USA) supplemented with 10% FBS was added, ensuring that half of the tissue blocks were submerged in the medium. The dishes were maintained at 37 °C in 5% CO_2_. On the following day, additional culture medium was added to slightly submerge the tissue blocks, and the medium was changed as needed based on cell growth. The TSCC cell line CAL27, obtained from ATCC (Rockville, MD, USA), was cultured in DF12 with 5% FBS. All cells were confirmed to be mycoplasma-free.

### 4.2. RNA Extraction and RT-PCR

Total RNA was extracted using Magzol reagent (#R4801-03, Magen, Shanghai, China) and reverse transcribed with the First Strand cDNA Synthesis Kit ReverTra Ace-α (#FSK-100, TOYOBO, Osaka, Japan). Target cDNA was amplified using the 2× Phanta Max Master Mix (Dye Plus) system (#P525-01, Vazyme, Nanjing, China) for 25–35 cycles in a total volume of 10 μL. The PCR products were loaded for agarose gel electrophoresis. Subsequently, all the PCR products were cloned into pEASY^®^-T1 Cloning Vector (#CT101-01, TransGen Biotech, Beijing, China) and verified by sequencing analysis. Target cDNA was also amplified with LightCycler^®^ 480 SYBR Green I Master (#04707516001, Roche, Mannheim, Germany) for qRT-PCR. Relative gene expression was calculated using the 2-ΔΔCt method, with GAPDH as the reference gene. The primer sequences are provided in Supplementary Table S3.

### 4.3. Cell Viability and Chemotherapeutic Sensitivity Assay

Cells were seeded in 96-well plates (5 × 10^3^ cells/well) and treated with different concentrations of CDDP for 48 h. Cell viability was measured using the CellTiter-Glo luminescent assay (#G7570, Promega, Madison, WI, USA) according to the protocol. Dose–response curves were generated with GraphPad Prism 9 (GraphPad Software Inc., San Diego, CA, USA).

### 4.4. Neosphere Formation Assay

Cells were seeded in agarose-coated 100 mm cell culture dishes (1 × 10^5^ cells/mL) in serum-free DF12 medium supplemented with 8 factors (8F), including 10 μg/mL human insulin, 5 μg/mL human transferrin, 10 μM 2-aminoethanol, 10 nM sodium selenite, 10 μM mercaptoethanol, BSA-oleid acid, heparin, and vitamin C. Spheres (diameter ≥ 50 µm) were counted after 6 days.

### 4.5. In Vivo Tumorigenicity Assay

Animal experiments were conducted in accordance with the Declaration of Helsinki and approved by the ethics committee of Sun Yat-sen University and the Institutional Animal Care and Use Committee (IACUC) (Ethical approval number: SYSU-IACUC-2022-001471). CTSC-1 or CTSC-2 cells were suspended in 50 µL of 1:1 DF12: Matrigel (#356234, BD Biosciences, Corning, NY, USA) and subcutaneously injected into the backs of female athymic nude mice. Female athymic BALB/c nude mice (4–6 weeks old) were purchased from Laboratory Animal Center of Sun Yat-sen University (Guangdong, China). Animals were euthanized after 3 months.

### 4.6. Hematoxylin–Eosin (H&E) Staining

The xenografts were harvested and fixed in 10% neutral formalin for 48 h and then sliced into 3–5 μm sections. After deparaffinization, sections were washed in 1 × PBS and then treated with hematoxylin for 5 min and washed in flow water for 10 min. They were then stained with eosin for 1 min and dehydrated in ethanol and xylene.

### 4.7. Plasmids and Viral Transduction

The pLenti-TP53-Q331*-FLAG and pLenti-TP53-WT-FLAG constructs were generated by cloning TP53 cDNAs into the pCDH-CMV-MCS-EF1-Zeocin vector (#72265, Addgene Plasmid, Cambridge, MA, USA, Revamped). The pLenti-ID2-FLAG constructs were generated by cloning the respective ID2 cDNAs into the pCDH-CMV-MCS-EF1-Zeocin vector. For CRISPR/Cas9-mediated gene knockout, guide RNAs targeting TP53 and ID2 were designed and cloned into the lentiCRISPRv2 vector (#52961, Addgene Plasmid, Cambridge, MA, USA). Lentiviral particles were produced by co-transfecting 293T cells with the lentiviral vector, psPAX2, and pMD2.G using Lipofectamine 2000 (#11668027, Thermo Fisher, Waltham, MA, USA). Viral supernatants were harvested 48 h after transfection, filtered through a 0.45 µm membrane, and used to infect cells in the presence of 8 µg/mL polybrene. Transduced cells were selected with 1 µg/mL puromycin after 48 h infection. Two independent single-cell knockout clones were obtained through single-cell sorting and expanded for downstream analysis, while data from representative clone are shown for clarity.

### 4.8. Flow Cytometry Analysis

CTSC-1 and CTSC-2 (1 × 10^6^ cells each) were fixed with 4% paraformaldehyde (PFA) for 15 min at room temperature, then incubated with cytokeratin 14 polyclonal antibody (#CL647-10143, Proteintech, Chicago, IL, USA) for 1 h at room temperature, and resuspended in 1× PBS. Beckman MoFlo Astrios EQs (Beckman, Brea, CA, USA) was used to sort and analyze cells. FlowJo-v1 software was used to analyze results.

### 4.9. Colony Formation ASSAY

Cells were seeded in 6-well plates (500 cells/well) for 8 days. The colonies were fixed with 4% PFA for 15 min at room temperature and stained with crystal violet (Sigma-Aldrich, St. Louis, MO, USA). More than three wells were prepared for each condition, and the colony-forming ability was assessed by counting the number of colonies.

### 4.10. In Vitro Differentiation Assay

The cells were seeded in 24-well plates (1 × 10^5^ cells/well) and cultured in adipogenic differentiation medium (#A1007001, Thermo Fisher, Waltham, MA, USA) for 30 days. Mature adipocytes were identified by Sudan III staining.

### 4.11. Western Blotting

Cells were lysed in RIPA buffer supplemented with protease inhibitor (#04693132001, Roche, Mannheim, Germany). The Pierce BCA protein quantification kit (#23225, Thermo Fisher, Waltham, MA, USA) was used to analyze the quantity of proteins. Equal amounts of protein were separated by SDS-PAGE, transferred to 0.45 μm PVDF membranes, and probed with primary antibodies against p53 (#2527T, Cell Signaling, Danvers, MA, USA), GAPDH (#60004-1-Ig, Proteintech, Chicago, IL, USA). Both anti-mouse and anti-rabbit secondary antibodies were from Abcam (#ab6721 and #ab205719, respectively, Abcam, Cambridge, UK), with marker #RM19001 (ABclonal, Wuhan, China).

### 4.12. RNA Sequencing and Bioinformatic Analysis

RNA sequencing (RNA-seq) was carried out by Novogene Bioinformatics Technology Co., Ltd. (Beijing, China). Total RNA was extracted from the samples using the RNeasy Mini Kit (#74104, Qiagen, Hilden, Germany). The integrity and quality of the total RNA, as well as the constructed transcriptome libraries, were assessed using the Agilent 2100 Bioanalyzer (Agilent, Santa Clara, CA, USA). Library construction was performed following Illumina’s protocols, and sequencing was carried out on the Illumina HiSeq™ 2000 platform (Illumina, San Diego, CA, USA). Raw sequencing reads underwent quality control. Gene expression levels were quantified with HTSeq, yielding raw, unnormalized integer counts for each gene. TPM (transcripts per kilobase million) values were computed for visualization. Differential gene expression was analyzed using the DESeq2_1.46.0 software package (Chapel Hill, NC, USA), which internally normalizes the raw counts to account for differences in sequencing depth and library composition. Differentially expressed genes were identified based on statistical testing with thresholds set at an adjusted *p*-value (padj) < 0.05 and |log2(FoldChange)| > 2. Functional annotation and KEGG pathway enrichment analysis of the significant differentially expressed genes were performed to identify enriched pathways. The sequencing data were deposited in the NCBI Sequence Read Archive (SRA) database under the BioProject accession number PRJNA1212792.

### 4.13. Luciferase Reporter Assay

ID2 promoter fragments (−2000 to +100 relative to TSS) were PCR-amplified from human genomic DNA and cloned into the pGL3-basic luciferase reporter vector (#E1741, Promega, Madison, WI, USA). Mutation of p53 response elements was performed with the QuikChange II XL Site-Directed Mutagenesis Kit (#200521, Agilent, Santa Clara, CA, USA). Cells were co-transfected with reporter constructs (wild type or mutant) and pRL-TK Renilla luciferase control plasmid (#D2760, Beyotime, Shanghai, China) using Lipofectamine 2000. After 48 h, luciferase activity was measured with the Dual-Luciferase Reporter Assay System (#E1910, Promega, Madison, WI, USA) and normalized to Renilla luciferase activity.

### 4.14. Clinical Datasets and Statistical Analysis

Publicly available datasets were analyzed by UALCAN and cBioPortal. https://ualcan.path.uab.edu/cgi-bin/ualcan-res.pl (accessed on 12 December 2024). https://www.cbioportal.org/ (accessed on 12 December 2024).

Statistical significance was determined using two-tailed unpaired Student’s *t*-tests and one-way ANOVA or two-way ANOVA, using GraphPad Prism 9 (GraphPad Software Inc., San Diego, CA, USA). A *p*-value < 0.05 was considered statistically significant.

## Data Availability

All the required data are provided with the manuscript in terms of figures, tables, and supplementary figures. The RNA sequencing data have been uploaded to NCBI; the accession to cite for the SRA data is PRJNA1212792, http://www.ncbi.nlm.nih.gov/bioproject/1212792 (accessed on 3 March 2025).

## Supplementary Materials

The following supporting information can be downloaded at: https://www.mdpi.com/article/10.3390/ijms26052353/s1.
