# Supplementary material for: A Truncated Mutation of TP53 Promotes Chemoresistance in Tongue Squamous Cell Carcinoma
Source: Int J Mol Sci. 2025 Mar 6;26(5):2353. doi: 10.3390/ijms26052353 (PMC11900931; doi:10.3390/ijms26052353)
Supplement: Supplementary file 1 [file ijms-26-02353-s001.zip › Supplemental table 1-STR.pdf]

	CTSC-1 specimen	CTSC-1 cell	CTSC-2 specimen	CTSC-2 cell
Marker	Allele		Allele	
D3S1358	15	15	15,16	15,16
TH01	7,9	7,9	7	7
D21S11	29	29	32.2,33.2	32.2,33.2
D18S51	14,15	14,15	12,15	15
Penta_E	15,16	15,16	19,20	19,20
D5S818	9,10	9,10	13,14	13,14
D13S317	11	11	8,9	8,9
D7S820	10,12	10,12	11,13	11,13
D16S539	9,10	10	9	9
CSF1PO	10,12	10,12	12	12
Penta_D	9,11	9	7,9	7,9
vWA	16,17	16,17	14,18	14,18
D8S1179	10,13	10,13	12,15	12,15
TPOX	8,11	8,11	8,9	8,9
FGA	19,24	24	20,25	20,25
Aml	X	X	X	X
D18S1364	16	16	16,18	18
D12S391	20,21	20,21	18,19	18,19
D13S325	19,20	19,20	19,20	19,20
D6S1043	11,14	11,14	14,18	14,18
D2S1772	19,24	19,24	21,24	21,24
D11S2368	18,19	18,19	18,19	18,19
D22-GATA198B05	14,21	14,21	17,21	17,21
D8S1132	20,22	20,22	20,21	20,21
D7S3048	23,24	23,24	20	20
