# Supplementary material for: A Truncated Mutation of TP53 Promotes Chemoresistance in Tongue Squamous Cell Carcinoma
Source: Int J Mol Sci. 2025 Mar 6;26(5):2353. doi: 10.3390/ijms26052353 (PMC11900931; doi:10.3390/ijms26052353)
Supplement: Supplementary file 1 [file ijms-26-02353-s001.zip › Supplemental table 2 RNA-seq mutations.pdf]

Gene	Mutation	Cell type
TP53	Q331*	CTSC-1
	P72R	CTSC-1 / CTSC-2
NOTCH1	D1698E	CTSC-1 / CTSC-2
	D2185E	CTSC-1 / CTSC-2
	N104M	CTSC-1 / CTSC-2
FAT1	H1273P	CTSC-1 / CTSC-2
	N1662S	CTSC-1 / CTSC-2
PTEN	T50 Delete	CTSC-1 / CTSC-2
CASP8	K14R	CTSC-1 / CTSC-2
TGFBR2	M1 Delete	CTSC-1 / CTSC-2
