# Supplementary material for: A Truncated Mutation of TP53 Promotes Chemoresistance in Tongue Squamous Cell Carcinoma
Source: Int J Mol Sci. 2025 Mar 6;26(5):2353. doi: 10.3390/ijms26052353 (PMC11900931; doi:10.3390/ijms26052353)
Supplement: Supplementary file 1 [file ijms-26-02353-s001.zip › Supplemental table 3-primer.pdf]

Name of primer		Sequence (5’ to 3’)
TP53	F	GCAGTCAGATCCTAGCGTCG
	R	AGTCTGAGTCAGGCCCTTCT
overexpression -TP53	F	CGGAATTCCGAATGTCAGTCTGAGTCAGGCC
	R	CGGGATCCCGCATGGAGGAGCCGCAGTC
sg TP53	F	CACCGGGATGATTTGATGCTGTCCC
	R	AAACGGGACAGCATCAAATCATCCC
ID2	F	AAAAACAGCCTGTCGGACCA
	R	TGCAGGCTGACAATAGTGGG
overexpression - ID2	F	GCTCTAGAGCATGAAAGCCTTCAGTCCCGTG
	R	CGGGATCCCGGCCACACAGTGCTTTGCTG
sg ID2	F	CACCGTGCCCAGGCTGTGGTCCGAC
	R	AAACGTCGGACCACAGCCTGGGCAC
ERCC4	F	GGAGTTGAACACCTCCCTCG
	R	GCTCTATACACCAAGATGCCAGTAAT
ERCC8	F	GCAGTGTGTTCCATTGGCAG
	R	GTGCTTGGTGGAGACTGGAG
ID2-promoter	F	CTAGCTAGCTAGTAATGCCTCCCGCGCTG
	R	CCCAAGCTTGGGGTCCAGGGCGATCTGCAG
Sox2	F	AGCTACAGCATGATGCAGGA
	R	GGTCATGGAGTTGTACTGCA
Bmi-1	F	CCACCTGATGTGTGTGCTTTG
	R	TTCAGTAGTGGTCTGGTCTTGT
Lgr5	F	CTCCCAGGTCTGGTGTGTTG
	R	GAGGTCTAGGTAGGAGGTGAAG
β-actin	F	TGCGCCGTTCCGAAAGTT
	R	GCGCCGCTGGGTTTTATAG
