# Supplementary material for: A Truncated Mutation of TP53 Promotes Chemoresistance in Tongue Squamous Cell Carcinoma
Source: Int J Mol Sci. 2025 Mar 6;26(5):2353. doi: 10.3390/ijms26052353 (PMC11900931; doi:10.3390/ijms26052353)
Supplement: Supplementary file 1 [file ijms-26-02353-s001.zip › Supplementary Figures and Figure Legends.pdf]

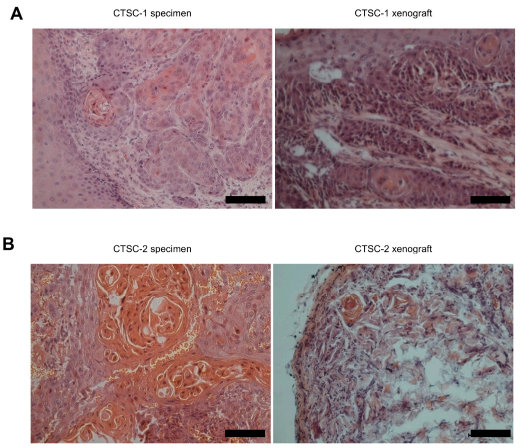

Supplementary Figure 1. Tumor histological features of CTSC-1 and CTSC-2. (A, B) H&E staining of tumors from CTSC-1 and CTSC-2 specimen and xenograft. Scale bars = 100 μm.

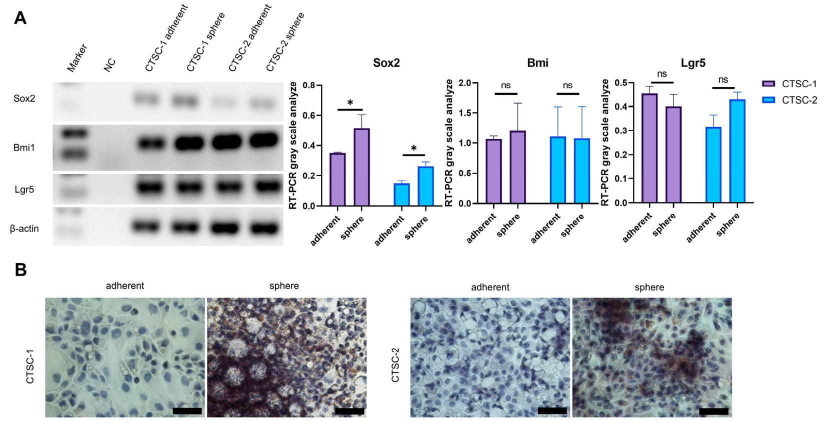

Supplementary Figure 2. CTSC-1 and CTSC-2 cells have different cellular characteristics on stemness. (A) RT-PCR was used to detect the expression of stem cell markers Sox2, Bmi1 and Lgr5 in adherent and sphere cells of CTSC-1 and CTSC-2 and gray scale analysis. (B) Microscopic images of adipocyte induction of CTSC-1 and CTSC-2 adherent and sphere cells. Scale bars = 50 μm.

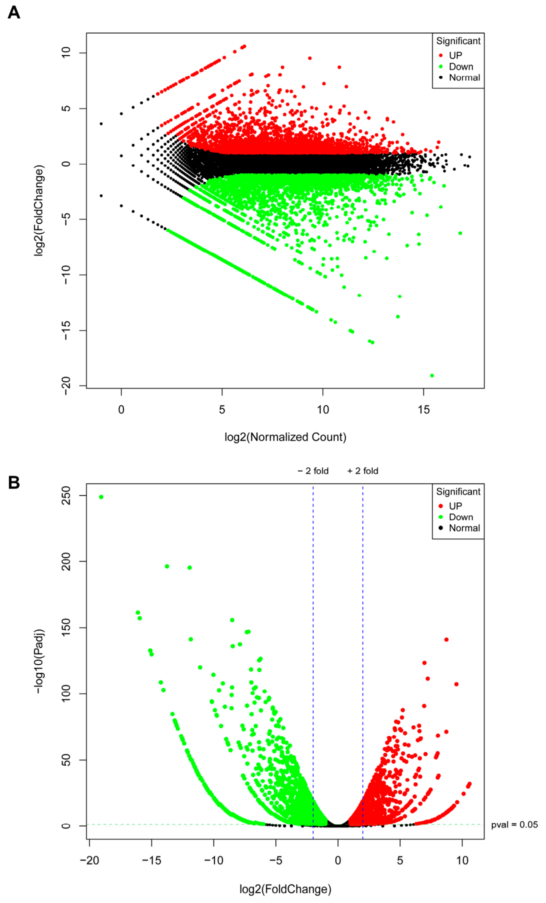

Supplementary Figure 3. Data analysis was performed using DESeq2, and MA plot and volcano plot were made to visualize gene expression changes.

(A) The MA plot shows the difference in gene expression between CTSC-1 and CTSC-2. (B) Volcano plot is used to display the magnitude (Fold Change) and statistical significance (p-value) of gene expression changes, and to identify genes with significant differences.

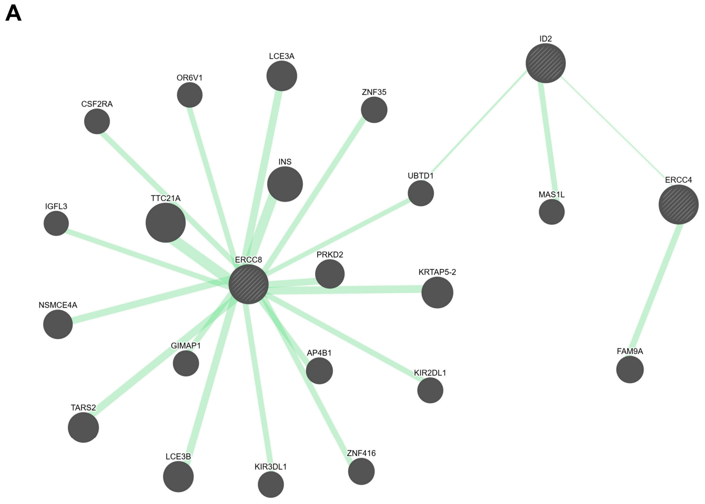

Supplementary Figure 4. The interaction network between ID2 and ERCC4/8.
